# Effect of exenatide on the cardiac expression of adiponectin receptor 1 and NADPH oxidase subunits and heart function in streptozotocin-induced diabetic rats

**DOI:** 10.1186/1758-5996-6-29

**Published:** 2014-02-28

**Authors:** Zhixin Guo, Wei Qi, Yuanxian Yu, Shijing Du, Jieping Wu, Jinjin Liu

**Affiliations:** 1 Department of Endocrinology, Second Hospital, Shanxi Medical University, 382 Wuyi Road, Taiyuan 030001, Shanxi, P.R China

**Keywords:** Exenatide, Diabetic cardiomyopathy, Adiponectin receptor 1, Glucose transporter type 4, NADPH oxidase

## Abstract

**Background:**

This study investigated the effect of exenatide on the cardiac expression of adiponectin receptor 1 and nicotinamide adenine dinucleotide phosphate (NADPH) oxidase subunits and heart function in streptozotocin-induced diabetic rats.

**Methods:**

Male Sprague–Dawley rats were randomly divided into four groups, i.e. control group, diabetic group, diabetic treated with low doses of exenatide (2 μg · kg^−1^.d^−1^) and diabetic treated with high doses of exenatide (10 μg · kg^−1^.d^−1^). Diabetes was induced by intraperitoneal injection of streptozotocin (65 mg/kg body weight). At the termination after exenatide treatment for eight weeks, following anesthesia of the rats, a catheter was inserted into the left ventricle through the right common carotid artery for measurement of left ventricular pressure, which included left ventricular systolic pressure (LVSP), left ventricular end-diastolic pressure (LVEDP) and the maximal rate of rise and decline of ventricular pressure (±dp/dt[max]). Plasma and myocardial adiponectin levels, and the expressions of myocardial adiponectin receptor 1, p22phox, NADPH oxidase 4 (NOX4), glucose transporter type 4 (Glut4), AMPK-α, phosphorylated-AMPK-α, connective tissue growth factor (CTGF) and copper zinc superoxide dismutase (Cu-Zn-SOD) were assayed.

**Results:**

Heart function, plasma adiponectin levels, the protein expression of myocardial phosphorylated-AMPK-α, the mRNA expression of myocardial Glut4, and the positive expression of myocardial Cu-Zn-SOD were significantly decreased in diabetic. The protein expression of myocardial adiponectin receptor 1, the mRNA expression of myocardial p22phox and NOX4, and the positive expression of myocardial CTGF were significantly increased in diabetic. Low and high doses of exenatide treatment significantly attenuated these changes in diabetic rats.

**Conclusions:**

These results suggest that exenatide may contribute to the improvement of the heart function in diabetic rats by down-regulating the expression of myocardial adiponectin receptor 1, p22phox and NOX4, and up-regulating plasma adiponectin level and the expression of myocardial AMPK-α, Glut4 and Cu-Zn-SOD.

## Background

Cardiovascular disease is one of the major complications of diabetes, resulting in a high risk of morbidity and mortality and causing significant burden for the healthcare system [1]. Glucagon-like peptide-1 (GLP-1) is an incretin hormone secreted by intestinal L-cells in response to nutrient ingestion [2]. The main physiological role of GLP-1 is to improve glycemic control by inducing glucose-dependent stimulation of insulin secretion and suppressing glucagon secretion [2]. Recently, increasing evidences indicate that GLP-1 may play a wide range of cardiovascular protective roles mediated by GLP-1 receptor in cardiovascular tissues [3-6].

Adiponectin and its receptors expressed in the heart may have important functions in the regulation of the cardiac function and/or metabolism [7,8]. Our previous studies [9-12] showed that the expression of myocardial adiponectin receptors were increased in type 1 diabetic rats and decreased in type 2 diabetic rats. Telmisartan and rosiglitazone treatment improved the heart function by upregulating the expression of adiponectin receptors in the heart of type 2 diabetic rats. It is unknown whether GLP-1 treatment can improve the heart function by regulating the expression of adiponectin receptors in the heart of diabetic rats.

Oxidative stress has been suggested to be involved in the development and progression of diabetes-induced cardiomyopathy [13]. Activation of nicotinamide adenine dinucleotide phosphate (NADPH) oxidase seems to be involved in the elevated oxidative stress in diabetes [14]. NADPH oxidase consists of the membrane-associated subunits (gp91phox and p22phox) and the cytosolic subunits (p47phox, p40phox, p67phox and Rac) [15]. Our previous studies [10,11,16] showed that the expression of NADPH oxidase subunits were increased in the heart of diabetic rats. N-acetylcysteine, telmisartan and rosiglitazone treatments inhibit the myocardial expression of NADPH oxidase subunits, which may result in the improvement of heart function in diabetic rats [10,11,16]. It is unknown whether GLP-1 treatment can downregulate the expression of NADPH oxidase subunits, p22phox and NOX4, in the heart of diabetic rats.

The primary aim of this study was to explore whether GLP-1 produced protective effects on the heart of diabetic rats independent of its glycemic control effect. The secondary aim of this study was to investigate the effect of GLP-1 on the expression of adiponectin receptors and NADPH oxidase subunits in the heart of a diabetic rat model.

## Methods

### Induction of diabetes

Forty-two male Sprague–Dawley rats aged 6 weeks, weighing 140-180 g, and purchased from the experimental animal center of Shanxi Medical University (Taiyuan, Shanxi, China), were used in the study ([Batch number of rats: SCXK (Jin) 2009–0001)]. All rats were housed in a temperature-controlled room (22-24°C) and kept on 12/12 hour light/dark cycles. They had free access to standard rat chow and water. All animals received humane care in accordance with the principles of the Chinese Council on Animal Care and the experimental protocol was approved by the Institutional Animal Care and Use Committee of Shanxi Medical University of China. After one week’s adaptation, all rats were randomly divided into 2 groups: a control group (C, n = 7) and a diabetic group (n = 35). Diabetic rats were given the peritoneal injection of a single dose of streptozotocin (65 mg/kg body weight; Sigma, St. Louis, MO, USA), while the control group was given equivalent volume of citric acid buffer. At 72 hr after streptozotocin injection, blood glucose was tested and rats with glucose levels greater than 16.7 mmol/L were considered as diabetic. There were 29 rats induced of diabetes. Diabetic rats were randomly divided into the diabetic group (D, n = 10), diabetic treated with low dose of exenatide (DTL, n = 10) and diabetic treated with high dose of exenatide (DTH, n = 9). Rats treated with low dose of exenatide were injected subcutaneously with exenatide in dose of 1 μg · kg^−1^ twice daily for 8 weeks. Rats treated with high dose of exenatide were injected subcutaneously with exenatide in the dose of 5 μg · kg^−1^ twice daily for 8 weeks. Control and diabetic rats were injected subcutaneously with equivalent volume of normal saline for 8 weeks. On 1 day before the experiments were terminated, all animals were weighed, fasted for 12–14 h, and then anesthetized with an intraperitoneal injection of 10% chloral hydrate (0.3 mL/100 g body weight), with efforts taken to reduce animal suffering. The heart function was measured by carotid artery cannula. The rats were sacrificed after blood sample had been drawn from abdominal cardinal vein. The blood samples were centrifuged and plasma aliquots were stored at −80°C until assays were done. The heart was immediately taken out of thoracic cavity after the rat was sacrificed. The heart was rinsed with normal saline, dried with filter-paper, and then weighed. The ratio of heart weight to body weight was calculated. The apex of heart was fixed in 10% neutral buffered formalin and processed for histological analysis. The rest part of cardiac ventricle was immediately placed into the liquid nitrogen and then stored in the –80°C refrigerator until analyses were performed.

### Measurement of cardiac function

Following anesthesia of the rats with an intraperitoneal injection of 10% chloral hydrate (0.3 ml/100 g body weight), the neck skin was cut open and the right common carotid artery was fully exposed. A micromanometer-tipped catheter was inserted into the left ventricle through the right common carotid artery for measurement of left ventricular pressure. Left ventricular systolic pressure (LVSP), left ventricular end-diastolic pressure (LVEDP) and the maximal rate of rise and decline of ventricular pressure (±dp/dt[max]) were obtained by BL-410 Bio-signal analysis system (Chengdu TME Technology Co. Ltd, Sichuan, China).

### Plasma analytical procedures

The plasma glucose level was measured by the glucose oxidase method. Plasma cholesterol, triglyceride and free fatty acid were measured colorimetrically using commercially available kit (Zhejiang Dong’ou Diagnostic Products Co. Ltd., Zhejiang, China; Nanjing Built Technology Co. Ltd., Jiangsu, China). Plasma adiponectin was measured using a commercially available ELISA kit (Westang Biotechnology Co., Ltd, Shanghai, China). Plasma insulin was measured using a commercially available radioimmunoassay kit (Beijing Kemei Biological Technology Co., Ltd., Beijing, China).

### Determination of cardiac adiponectin

Frozen heart tissue was pulverized and homogenized at 4°C in cold buffer (20 mM Tris–HCl, pH 7.5, 50 mM 2-mercaptoethanol, 5 mM EGTA, 2 mM EDTA, 1 mM PMSF, 10 mM NaF, 25 μg/ml leupeptin, 2 μg/ml aprotinin) and then centrifuged at 1500 g for 5 minutes at 4°C. The supernatant was collected and stored at −80°C until analyses were conducted. The protein content of the samples was measured using the Bradford protein assay [17] with the use of bovine serum albumin as the concentration standard. Cardiac adiponectin was measured using a commercially available enzyme-linked immunosorbent assay (ELISA) kit (Westang Biotechnology Co., Ltd, Shanghai, China).

### Morphological study

Tissues fixed in 10% buffered formalin were embedded in paraffin, sectioned at 4 μm thickness and stained with hematoxylin and eosin (HE) for light microscopic morphological study.

### Immunohistochemical analysis of adiponectin receptor 1, CTGF and Cu-Zn-SOD

Ventricular samples were immediately fixed in 10% neutral buffered formalin overnight and embedded in paraffin. Paraffin embedded tissue blocks were sectioned at 3 μm and sections were mounted on positively charged slides. The slides were deparaffinized, rehydrated, blocked with 3% hydrogen peroxide [to block endogenous peroxidase activity, then washed with phosphate-buffered saline (PBS)], and blocked with 5% normal goat serum in PBS for 30 min. The slides were subsequently incubated with primary rabbit polyclonal adiponectin receptor 1 (1:400) antibody (Beijing Biosynthesis Biotechnology Co., Ltd, Beijing, China), connective tissue growth factor antibody (1:100, Wuhan Boster Biological Engineering Company Limited, Hubei, China), copper zinc superoxide dismutase (Cu-Zn-SOD) antibody (1:400, Wuhan Boster Biological Engineering Company Limited, Hubei, China) in PBS containing 1% normal goat serum overnight at 4°C. The primary antibody was rinsed off with PBS, and the sections were incubated with biotin labeling goat anti-rabbit secondary antibodies (1:100, Wuhan Boster Biological Engineering Company Limited, Hubei, China) for 30 min. After three washing steps in PBS were completed, the sections were stained using horseradish enzyme labeling strepto-avidin solution (1:100, Wuhan Boster Biological Engineering Company Limited, Hubei, China) for 10 min, washed with PBS, dyed with 3, 3′-diaminobenzidine (DAB), and washed with distilled water. The sections were counterstained using hematoxylin, washed in running water, dehydrated through a series of increasing alcohol concentrations, and cleared in xylene before being mounted in resinous mounting medium with coverslips. Some sections incubated with nonspecific rabbit immunoglobulins (IgG) served as negative controls. Quantification was performed with the observer blinded to details. With the use of high power microscope, all slides were observed and photographed. An average of five fields under microscope (×400) in each slice was randomly selected, and the average value of gray scale which had an inverse proportion to the positive stained intensity was quantified using BI-2000 color image processing system (Chengdu TME Technology Co., Ltd, Sichuan, China).

### Real-time fluorescence quantitative PCR analysis for p22phox, NOX4 and Glut4

Total RNA extraction was performed using Trizol reagent (Sangon Biotech Co., Ltd, Shanghai, China) according to the manufacturer’s recommendations. DNA was synthesized from total RNA and then was amplified using a RT-PCR reagent kit (Fermentas China Co., Ltd, Shenzhen, China). Real-time quantitative polymerase chain reaction was performed with 50 μl reaction volumes containing 25 μl 2 × FastStart Universal SYBR Green Master Mix (Roche Ltd., Basel, Switzerland), 1 μl 0.3 μM of each primer, 5 μl of cDNA template, and 19 μl deionized water. Primer sequences are described in Table 1. PCR amplification was done on a real-time fluorescence quantitative PCR detection system (Xi’an Tianlong Science and Technology Co., Ltd, Xi’an, China) using the following parameters: initial denaturation at 95°C for 10 min, followed by 40 cycles of denaturation at 94°C for 30 seconds, annealing at 58°C for 30 seconds, and extension at 72°C for 45 seconds. Relative gene expression levels were quantified using the comparative ΔCt (cycle threshold) method. This method normalizes Ct values of the detected gene to the average of that of the housekeeping genes and calculates the relative expression values as fold changes of the control. Data analysis was performed using the 2^-ΔΔCt^ method [18].

**Table 1:** Primers used in real-time fluorescence quantitative PCR

**Gene symbol**	**primer (5′ → 3′)**	**Product size (bp)**
Glut4		
Forward	5′- GATGCCGTCGGGTTTCCAGCA -3′	233 bp
Reverse	5′- TGAGGGTGCCTTGTGGGATGG -3′	
NOX4		
Forward	5′-TCTAGGCCGTAACGGAATTC-3′	245 bp
Reverse	5′-TGTAACCATGAGGAACAATACCACC-3′	
p22phox		
Forward	5′-CTCTATTGTTGCAGGAGTGC-3′	457 bp
Reverse	5′-TCACACGACCTCATCTGTCAC-3′	
β-actin		
Forward	5′-GTCAGGTCATCACTATCGGCAAT-3′	147 bp
Reverse	5′-AGAGGTCTTTACGGATGTCAACGT-3′	

Glut4: glucose transporter type 4.

### Western blotting analysis for adiponectin receptor 1, AMPK-α and phosphor-AMPK-α

Heart tissue (100 mg) was pulverized and homogenized using a Polytron homogenizer in 1.5 ml cold lysis buffer as described in Section 2.4. The homogenate was centrifuged at 15,000 g for 20 min at 4°C and the supernatant was collected. The protein content of the supernatant was measured using the Bradford protein assay [17]. Aliquot samples were stored at −80°C until use.

After boiling at 95°C for 5 min, samples (50 μg protein/lane) were loaded to 10-15% SDS-PAGE gel and then transferred to nitrocellulose membranes. The membranes were blocked in 5% non-fat milk in TBST (0.05% Tween-20 in 1 × Tris-buffered saline (TBS) and then incubated with primary antibodies of: adiponectin receptor 1 (1:1000) ( Santa Cruz Biotechnology Inc., Santa Cruz, CA, USA); AMPK-α and phosphor-AMPK-α (1:1000) (Cell Signalling Technology, Beverly, MA, USA). The membranes were washed and then incubated with a secondary horseradish peroxidase-conjugated anti-rabbit IgG antibody (Santa Cruz Biotechnology Inc., Santa Cruz, CA, USA). Proteins were visualized by 3, 3′-diaminobenzidin (DAB) (Zhongshan Goldenbridge Biotechnology Co., Ltd, Beijing, China) and quantified using image analysis software. In all instances, the membranes were stained with Ponceau stain and reblotted with antibody against β-actin (1:1000, Santa Cruz Biotechnology Inc., Santa Cruz, USA) after stripping to verify the uniformity of protein load and the transfer efficiency across the test samples.

### Statistical analysis

Data are expressed as means ± S.E.M. Statistical analysis was performed by one –way ANOVA followed by the Tukey’s post hoc test. P < 0.05 was considered statistically significant.

## Results

### Animal characteristics

The levels of plasma glucose, total cholesterol, triglycerides and free fatty acids were significantly increased, and the level of plasma insulin was significantly decreased in diabetic rats compared to controls (P<0.05). There was no significant difference in plasma glucose, total cholesterol, triglycerides, free fatty acids and insulin between diabetic rats treated with low dose of exenatide and untreated diabetic rats (P>0.05). The levels of plasma total cholesterol, triglycerides and free fatty acids were significantly decreased in diabetic treated with high dose of exenatide compared to diabetic untreated (P<0.05). However, there was no significant difference in plasma glucose and insulin between diabetic treated with high dose of exenatide and diabetic untreated (P>0.05). The levels of plasma free fatty acids were significantly decreased in diabetic treated with high dose of exenatide compared to diabetic treated with low dose of exenatide (P<0.05). However, there was no significant difference in plasma glucose, total cholesterol, triglycerides and insulin between diabetic treated with low dose of exenatide and diabetic treated with high dose of exenatide (P>0.05). The ratio of heart weight to body weight, as an index of cardiac hypertrophy, was significantly increased in diabetic rats compared to controls (P<0.05). Low or high doses of exenatide treatment did not attenuate the increased ratio of heart weight to body weight in diabetic rats (P>0.05) (Table 2).

**Table 2:** General characteristics in control, diabetic, and diabetic treated with low and high doses of exenatide

	**C**	**D**	**DTL**	**DTH**
Number	7	10	10	9
Plasma glucose (mM)	4.31 ± 0.72	29.86 ± 1.32^*^	29.08 ± 1.20^*^	28.66 ± 0.89^*^
Plasma Insulin (Iu/ml)	19.36 ± 0.58	4.91 ± 0.27^*^	5.06 ± 0.30^*^	5.19 ± 0.31^*^
Plasma total cholesterol (mM )	1.46 ± 0.38	2.79 ± 0.29^*^	2.77 ± 0.50^*^	2.27 ± 0.72a^*,#^
Plasma triglycerides (mM )	0.83 ± 0.13	1.42 ± 0.20^*^	1.29 ± 0.27^*^	1.08 ± 0.87^#^
Plasma free fatty acid (mM )	1.45 ± 0.20	2.60 ± 0.41^*^	2.26 ± 0.63^*^	1.53 ± 0.34^#,$^
Body weight (g)	459.57 ± 86.52	207.70 ± 48.89^*^	200.60 ± 74.48^*^	211.11 ± 41.20^*^
Heart weight (g)	1.30 ± 0.17	0.93 ± 0.29^*^	0.85 ± 0.20^*^	0.88 ± 0.23^*^
Heart/body weight (g/kg)	2.86 ± 0.38	4.50 ± 0.68^*^	4.42 ± 0.76^*^	4.42 ± 0.86^*^

Values were obtained at sacrifice, after administration with or without 8 weeks of exenatide treatment. Control (C), diabetic (D), diabetic treated with low dose (2 μg · kg^−1^.d^−1^) of exenatide (DTL), diabetic treated with high dose (10 μg · kg^−1^.d^−1^) of exenatide (DTH). All values were expressed as mean ± S.E.M. *P < 0.05 or 0.01 vs. C; ^#^P < 0.05 vs. D; ^$^P < 0.05 vs. DTL.

### Histological changes

**Figure 1:**
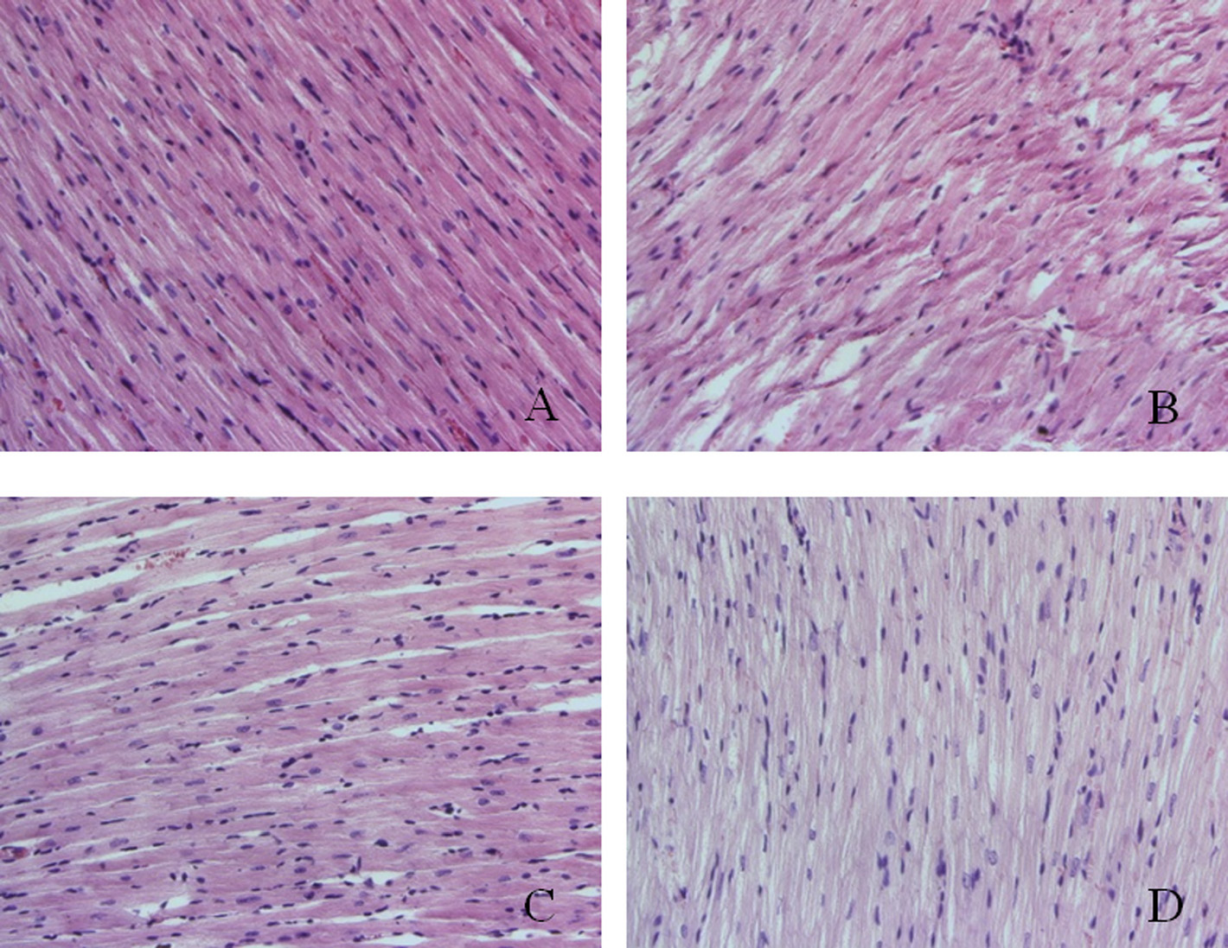
**Representative slides showing HE staining in the myocardium.** Slides **A**, **B**, **C**, **D** represent control, diabetic, diabetic treated with low dose (2 μg · kg^−1^.d^−1^) of exenatide and diabetic treated with high dose (10 μg · kg^−1^.d^−1^) of exenatide, respectively. Magnifications × 400.

The results of the myocardium stained by hematoxylin and eosin (HE) and observed through the light microscope in diabetic rats were as following: The myocardial cells lined up in order, the sizes of cellular nucleus were consistent, and the cytoplasm was stained in uniformity in control rats. The myocardial cells lined up in disorder, the sizes of cellular nucleus were inconsistent, and the myocardial fibers were broken and lined up in disorder in diabetic rats. The pathological changes were obviously alleviated in diabetic rats treated with low and high doses of exenatide than those in diabetic rats untreated. The myocardial cells lined up comparatively in order, the sizes of cellular nucleus were comparatively consistent, and there was no obviously broken myocardial fiber in diabetic rats treated with low and high doses of exenatide than those in diabetic rats untreated. The pathological changes were further alleviated in diabetic rats treated with high dose of exenatide compared to diabetic rats treated with low dose of exenatide (Figure 1).

### Heart function

Compared to controls, left ventricular systolic pressure (LVSP), +dp/dtmax and -dp/dtmax were significantly reduced and left ventricular end-diastolic pressure (LVEDP) was significantly increased in diabetic rats, indicating that the heart function was significantly decreased in diabetic rats. The high and low doses of exenatide treatment significantly attenuated these changes in diabetic rats. Compared to diabetic rats treated with low dose of exenatide, diabetic rats treated with high dose of exenatide had significant increased LVSP and decreased LVEDP (P<0.05). There was no significant difference in + dp/dtmax and -dp/dtmax between diabetic rats treated with low dose of exenatide and diabetic rats treated with high dose of exenatide (Table 3).

**Table 3:** Heart function, plasma and myocardial adiponectin levels in control, diabetic, and diabetic treated with low and high doses of exenatide

	**C**	**D**	**DTL**	**DTH**
Number	7	10	10	9
LVSP (mmHg)	142.48 ± 11.01	78.77 ± 14.56^*^	90.25 ± 10.67^*,#^	102.42 ± 9.32^*,#,$^
LVEDP (mmHg)	6.5 ± 1.42	11.2 ± 1.68^*^	9.58 ± 3.18^*,#^	7.28 ± 2.61^#,$^
+dp/dtmax (mmHg.s^−1^)	7780.51 ± 809.47	2182.12 ± 344.15^*^	3735.14 ± 384.41^*,#^	3892.61 ± 535.43^*,#^
-dp/dtmax (mmHg.s^−1^)	7014.15 ± 746.47	1838.39 ± 206.33^*^	3774.86 ± 419.87^*,#^	3997.33 ± 782.37^*,#^
Plasma adiponectin (ng/ml)	1877.96 ± 377.97	1032.33 ± 170.23^*^	1387.72 ± 343.71^#^	1396.82 ± 185.52^#^
Myocardial adiponectin (ng/μg protein)	0.13 ± 0.04	0.11 ± 0.03	0.13 ± 0.06	0.16 ± 0.05

Values were obtained at sacrifice, after administration with or without 8 weeks of exenatide treatment. Control (C), diabetic (D), diabetic treated with low dose (2 μg · kg^−1^.d^−1^) of exenatide (DTL), diabetic treated with high dose (10 μg · kg^−1^.d^−1^) of exenatide (DTH). LVSP: left ventricular systolic pressure; LVEDP: left ventricular end diastolic pressure; −dp/dtmax: the maximum descent-speed of pressure in isovolumic relaxation period in the left ventricle; +dp/dtmax: the maximum ascendent-speed of pressure in isovolumic contraction period in the left ventricle. All values were expressed as mean ± S.E.M. *P < 0.05 or 0.01 vs. C; ^#^P < 0.05 vs. D; ^$^P < 0.05 vs. DTL.

### Immunohistochemical assay

**Figure 2:**
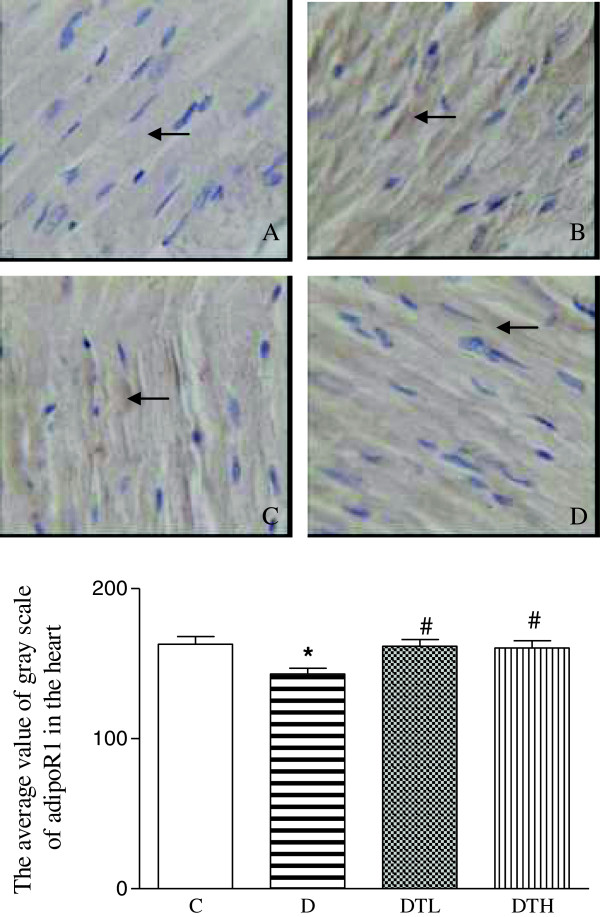
**Top panel: representative slides showing immunohistochemical staining of adiponectin receptor 1 (AdipoR1) (stained in brown as shown by arrow) in the myocardium.** Slides **A**, **B**, **C**, **D** represent control, diabetic, diabetic treated with low dose (2 μg · kg^−1^.d^−1^) of exenatide and diabetic treated with high dose (10 μg · kg^−1^.d^−1^) of exenatide, respectively. Magnifications × 400. Bottom: bar graph shows quantitative analysis of myocardial adipoR1 expression in control, diabetic and diabetic treated with low (2 μg · kg^−1^.d^−1^) or high (10 μg · kg^−1^.d^−1^) dose of exenatide. The adiponectin receptor 1 (adipoR1) expression was quantified using the average value of gray scale which had an inverse proportion to the positive stained intensity. Control (C, n = 7), diabetic (D, n = 10), diabetic treated with low dose (2 μg · kg^−1^.d^−1^) of exenatide (DTL, n = 10), diabetic treated with high dose (10 μg · kg^−1^.d^−1^) of exenatide (DTH, n = 9). Data are expressed as mean ± S.E.M. *P < 0.05 different from control, ^#^P < 0.05 different from diabetic.

The yellow positive staining of adiponectin receptor 1 was mainly located in myocardial cellular member and cytoplasm. The expression of adiponectin receptor 1 was quantified using the average value of gray scale which was inversely proportional to the positive staining intensity. The average value of gray scale of adiponectin receptor 1 was significantly decreased in the heart in diabetic rats compared to controls, indicating that the positive staining intensity of adiponectin receptor 1 was significantly increased in the heart in diabetic rats compared to controls. The high and low doses of exenatide treatment significantly increased the average value of gray scale of adiponectin receptor 1 in the heart in diabetic rats, indicating that exenatide treatment decreased the positive stained intensity of adiponectin receptor 1 in the heart in diabetic rats. There was no difference in the expression of cardiac adiponectin receptor 1 between diabetic rats treated with low dose of exenatide and diabetic rats treated with high dose of exenatide (Figure 2).

**Figure 3:**
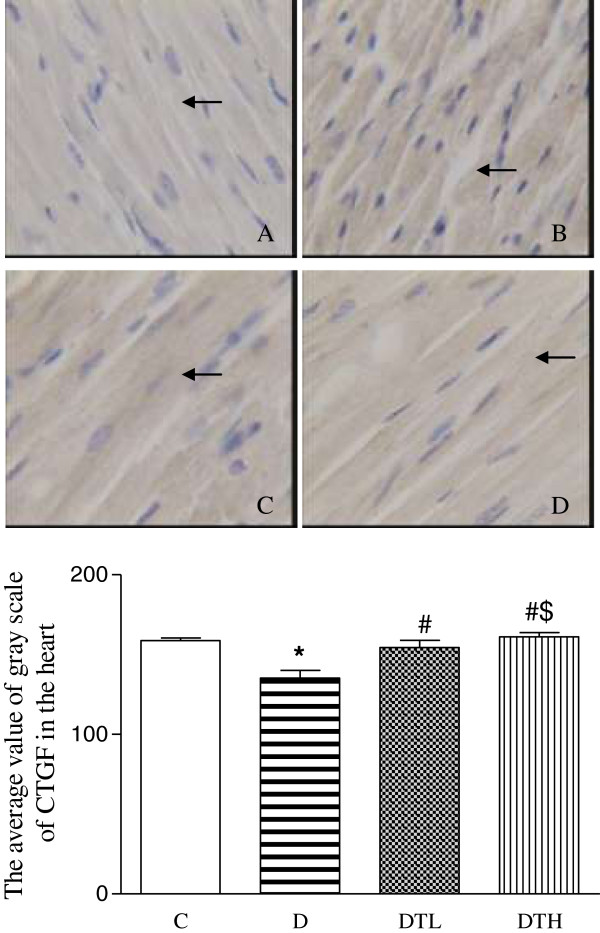
**Top panel: representative slides showing immunohistochemical staining of connective tissue growth factor (CTGF) (stained in brown as shown by arrow) in the myocardium.** Slides **A**, **B**, **C**, **D** represent control, diabetic, diabetic treated with low dose (2 μg · kg^−1^.d^−1^) of exenatide and diabetic treated with high dose (10 μg · kg^−1^.d^−1^) of exenatide, respectively. Magnifications × 400. Bottom: bar graph shows quantitative analysis of myocardial CTGF expression in control, diabetic and diabetic treated with low (2 μg · kg^−1^.d^−1^) or high (10 μg · kg^−1^.d^−1^) dose of exenatide. The CTGF expression was quantified using the average value of gray scale which had an inverse proportion to the positive stained intensity. Control (C, n = 7), diabetic (D, n = 10), diabetic treated with low dose (2 μg · kg^−1^.d^−1^) of exenatide (DTL, n = 10), diabetic treated with high dose (10 μg · kg^−1^.d^−1^) of exenatide (DTH, n = 9). Data are expressed as mean ± S.E.M. *P < 0.05 different from control, ^#^P < 0.05 different from diabetic, ^$^P < 0.05 different from diabetic treated with low dose (2 μg · kg^−1^.d^−1^) of exenatide.

The yellow positive staining of CTGF was mainly located in myocardial cellular cytoplasm. The CTGF expression was quantified using the average value of gray scale which was inversely proportional to the positive stained intensity. The average value of grey scale of CTGF was significantly decreased in diabetic rats compared to controls, indicating that the positive stained intensity of CTGF increased in diabetic rats compared to controls. The high and low doses of exenatide treatment significantly increased the average value of grey scale of CTGF in diabetic rats, indicating that exenatide treatment decreased the positive stained intensity of CTGF in diabetic rats. The average value of grey scale of CTGF was significantly increased in diabetic rats treated with high dose of enenatide compared to diabetic rats treated with low dose of enenatide, indicating that the positive stained intensity of CTGF decreased in high dose of enenatide treatment compared to low dose of enenatide treatment (Figure 3).

**Figure 4:**
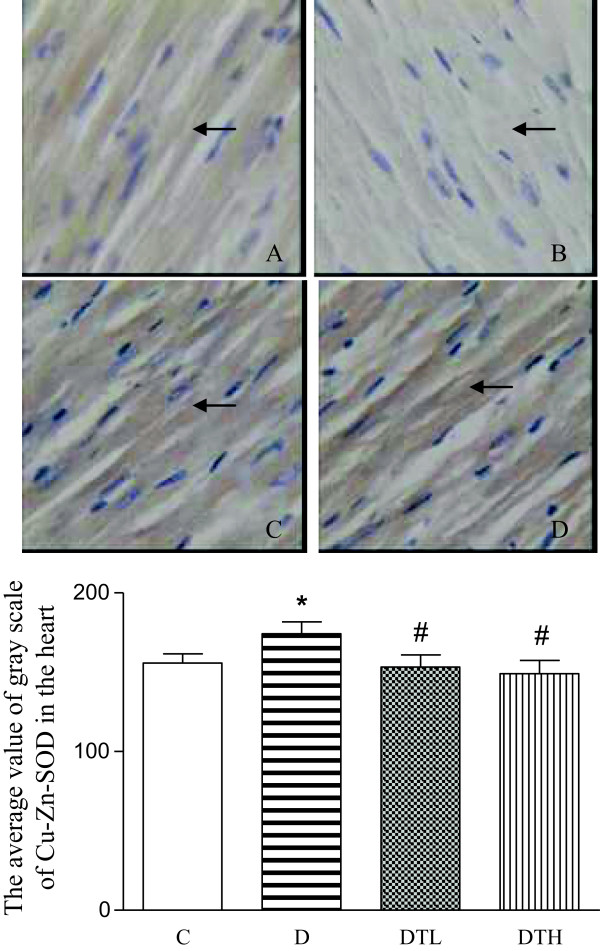
**Top panel: representative slides showing immunohistochemical staining of copper zinc superoxide dismutase (Cu-Zn-SOD) (stained in brown as shown by arrow) in the myocardium.** Slides **A**, **B**, **C**, **D** represent control, diabetic, diabetic treated with low dose (2 μg · kg^−1^.d^−1^) of exenatide and diabetic treated with high dose (10 μg · kg^−1^.d^−1^) of exenatide, respectively. Magnifications × 400. Bottom: bar graph shows quantitative analysis of myocardial Cu-Zn-SOD expression in control, diabetic and diabetic treated with low (2 μg · kg^−1^.d^−1^) or high (10 μg · kg^−1^.d^−1^) dose of exenatide. The Cu-Zn-SOD expression was quantified using the average value of gray scale which had an inverse proportion to the positive stained intensity. Control (C, n = 7), diabetic (D, n = 10), diabetic treated with low dose (2 μg · kg^−1^.d^−1^) of exenatide (DTL, n = 10), diabetic treated with high dose (10 μg · kg^−1^.d^−1^) of exenatide (DTH, n = 9). Data are expressed as mean ± S.E.M. *P < 0.05 different from control, ^#^P < 0.05 different from diabetic.

The yellow positive staining of Cu-Zn-SOD was mainly located in myocardial cellular cytoplasm. The Cu-Zn-SOD expression was quantified using the average value of gray scale which was inversely proportional to the positive stained intensity. The average value of gray scale of Cu-Zn-SOD was significantly increased in the heart in diabetic rats compared to controls, indicating that the positive stained intensity of Cu-Zn-SOD decreased in the heart in diabetic rats compared to controls. The high and low doses of exenatide treatment significantly decreased the average value of gray scale of Cu-Zn-SOD in the heart in diabetic rats, indicating that exenatide treatment increased the positive stained intensity of Cu-Zn-SOD in the heart in diabetic rats. There was no difference in the expression of cardiac Cu-Zn-SOD between diabetic rats treated with low dose of exenatide and diabetic rats treated with high dose of exenatide (Figure 4).

### Plasma and myocardial adiponectin levels

Plasma adiponectin levels were significantly decreased in diabetic rats compared to controls. The high and low doses of exenatide treatment significantly increased plasma adiponectin levels in diabetic rats. There was no significant difference in plasma adiponectin levels between diabetic rats treated with low dose of exenatide and diabetic rats treated with high dose of exenatide. There was no significant difference in the myocardial adiponectin levels among the four groups (Table 3).

### Myocardial protein expression of adiponectin receptor 1

**Figure 5:**
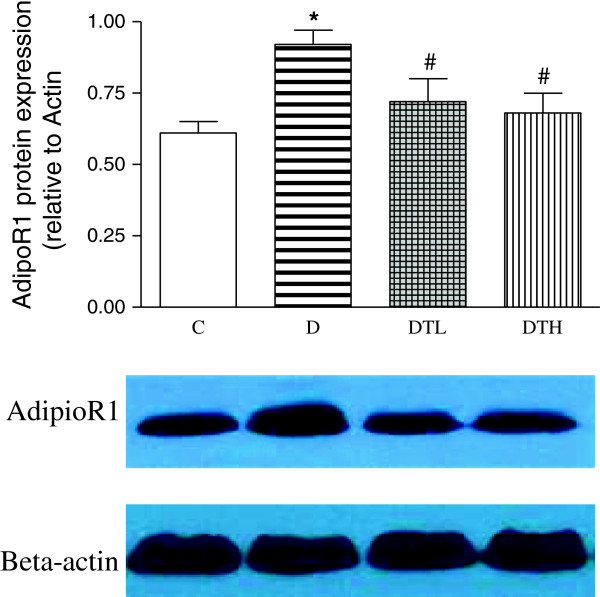
**Western blot analysis of myocardial protein expression of adiponectin receptor 1 (AdipoR1) in control, diabetic and diabetic treated with low (2 μg · kg**^**−1**^**.d**^**−1**^**) or high dose (10 μg · kg**^**−1**^**.d**^**−1**^**) of exenatide.** Equal protein loading was confirmed with β-actin. Mean band density was normalized relative to β-actin. Control (C, n = 7), diabetic (D, n = 10), diabetic treated with low dose (2 μg · kg^−1^.d^−1^) of exenatide (DTL, n = 10), diabetic treated with high dose (10 μg · kg^−1^.d^−1^) of exenatide (DTH, n = 9). Data are expressed as mean ± S.E.M. *P < 0.05 different from control, ^#^P < 0.05 different from diabetic.

The protein expression of myocardial adiponectin receptor 1 significantly increased in diabetic rats compared to controls. The high and low doses of exenatide treatment significantly decreased the protein expression of myocardial adiponectin receptor 1 in diabetic rats. There was no difference in the protein expression of myocardial adiponectin receptor 1 between diabetic rats treated with low dose of exenatide and diabetic rats treated with high dose of exenatide (Figure 5).

### Myocardial phospho-AMPK-alpha (Thr172) protein expression

**Figure 6:**
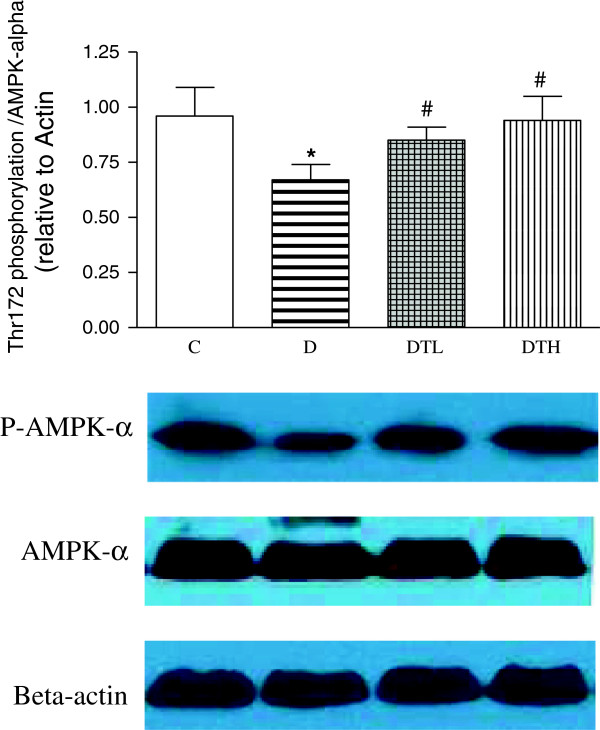
**Western blot analysis of renal protein expression of phospho-AMPK- alpha (Thr172) and AMPK-alpha in control, diabetic and diabetic treated with low (2 μg · kg**^**−1**^**.d**^**−1**^**) or high (10 μg · kg**^**−1**^**.d**^**−1**^**) dose of exenatide.** Equal protein loading was confirmed with β-actin. Mean band density was normalized relative to β-actin. Control (C, n = 7), diabetic (D, n = 10), diabetic treated with low dose (2 μg · kg^−1^.d^−1^) of exenatide (DTL, n = 10), diabetic treated with high dose (10 μg · kg^−1^.d^−1^) of exenatide (DTH, n = 9). Data are expressed as mean ± S.E.M. *P < 0.05 different from control, ^#^P < 0.05 different from diabetic.

The level of myocardial phosphorylation of AMPK-alpha (Thr172) was significantly decreased in diabetic rats compared to controls. The high and low doses of exenatide treatment significantly increased the level of myocardial phosphorylation of AMPK-alpha (Thr172) in diabetic rats. There was no difference in the level of myocardial phosphorylation of AMPK-alpha (Thr172) between diabetic rats treated with low dose of exenatide and diabetic rats treated with high dose of exenatide (Figure 6).

### Myocardial mRNA expression of Glut4

**Figure 7:**
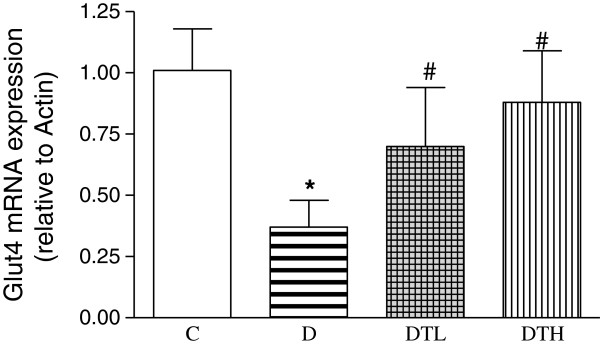
**Real-time fluorescence quantitative polymerase chain reaction (PCR) analysis of myocardial mRNA expression of glucose transporter type 4 (Glut4) in control, diabetic and diabetic treated with low (2 μg · kg**^**−1**^**.d**^**−1**^**) or high (10 μg · kg**^**−1**^**.d**^**−1**^**) dose of exenatide.** Relative gene expression levels were normalized relative to β-actin. Control (C, n = 7), diabetic (D, n = 10), diabetic treated with low dose (2 μg · kg^−1^.d^−1^) of exenatide (DTL, n = 10), diabetic treated with high dose (10 μg · kg^−1^.d^−1^) of exenatide (DTH, n = 9). Data are expressed as mean ± S.E.M. *P < 0.05 different from control, ^#^P < 0.05 different from diabetic.

The mRNA expression of myocardial Glut4 was significantly decreased in diabetic rats compared to controls. The high and low doses of exenatide treatment significantly increased the mRNA expression of myocardial Glut4 in diabetic rats. There was no difference in the mRNA expression of myocardial Glut4 between diabetic rats treated with low dose of exenatide and diabetic rats treated with high dose of exenatide (Figure 7).

### Myocardial mRNA expression of p22phox and Nox4

**Figure 8:**
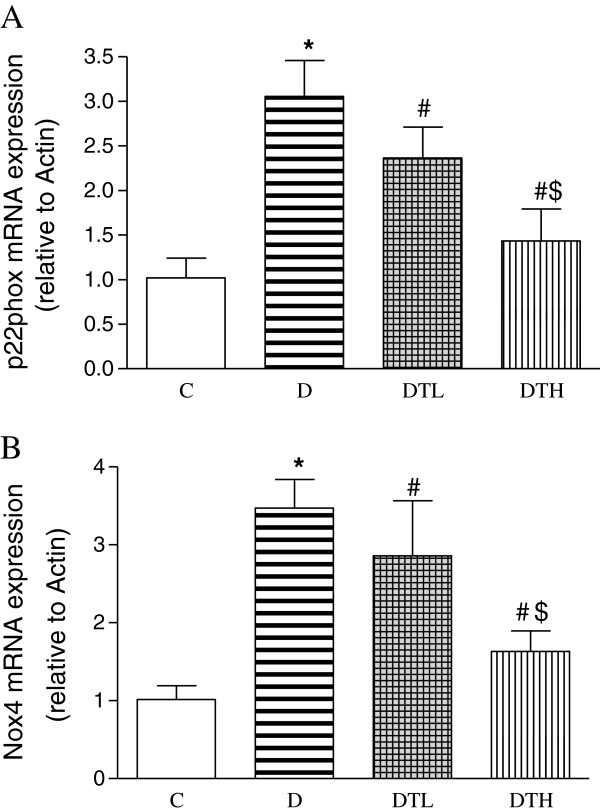
**Real-time fluorescence quantitative polymerase chain reaction (PCR) analysis of myocardial mRNA expression of p22phox (A) and NOX4 (B) in control, diabetic and diabetic treated with low (2 μg · kg**^**−1**^**.d**^**−1**^**) or high (10 μg · kg**^**−1**^**.d**^**−1**^**) dose of exenatide.** Relative gene expression levels were normalized relative toβ-actin. Control (C, n = 7), diabetic (D, n = 10), diabetic treated with low dose (2 μg · kg^−1^.d^−1^) of exenatide (DTL, n = 10), diabetic treated with high dose (10 μg · kg^−1^.d^−1^) of exenatide (DTH, n = 9). Data are expressed as mean ± S.E.M. *P < 0.05 different from control, ^#^P < 0.05 different from diabetic, ^$^P < 0.05 different from diabetic treated with low dose (2 μg · kg^−1^.d^−1^) of exenatide.

The mRNA expression of myocardial p22phox and Nox4 was significantly increased in diabetic rats compared to controls. The high and low doses of exenatide treatment significantly decreased the mRNA expression of myocardial p22phox and Nox4 in diabetic rats. The mRNA expression of myocardial p22phox and Nox4 was significantly decreased in diabetic rats treated with high dose of enenatide compared to diabetic rats treated with low dose of enenatide (Figure 8).

## Discussion

In the present study, we showed that exenatide exerted protective effects on the heart through antioxidative actions and regulation effect on adiponectin and its receptors without lowering the blood glucose level in a streptozotocin-induced rat model of type 1 diabetes. This is the first report of a GLP-1 receptor agonist may contribute, via its regulation effect on adiponectin receptors, to amelioration of heart function in diabetic rats.

Glucagon like peptide 1 (GLP-1) is one of the incretin secreted by intestinal L cell. GLP-1 receptor is distributed widely across different tissues. In addition to the pancreas, GLP-1 receptor is also expressed in the lung, brain, kidney, and heart, etc. [19]. In the heart, GLP-1 receptor is distributed in the cardiomyocyte, endocardium, microvascular endothelium and coronary smooth muscle cells. GLP-1 receptor plays an important role in maintaining the normal myocardial structure and physiological function [20]. Mice with genetic deletion of the GLP-1 receptor (GLP-1R (−/−)) exhibit elevated left ventricular end diastolic pressure, increased left ventricular thickness, and impaired left ventricular contractility and diastolic function, indicating that an essential role for GLP-1 receptor in the control of murine cardiac structure and function *in vivo*[21]. Many studies showed that GLP-1 improved heart function and played a protective role on the cardiovascular system by binding with the GLP-1 receptor expressed in the cardiovascular tissue [21-27]. Our study showed that the levels of blood glucose and lipids were significantly increased and the heart function was deteriorated in diabetic rats. GLP-1 receptor agonist exenatide decreased the level of blood lipids, improved the heart function, and alleviated the myocardial pathological lesion, but did not affect the level of blood glucose, suggesting that GLP-1 receptor agonist could produce the protective effect on the heart of diabetic rats independent of its hypoglycemic effect. The reason why exenatide had no effect on reducing blood glucose in type 1 diabetic rats in the current study was that exenatide did not restore insulin secretion in our model as shown in Table 2. Our results are in agreement with the results of a previous study [28] showing that GLP-1 receptor agonist had no effect on reducing blood glucose in a rat model of type 1 diabetes.

Adiponectin is an adipocyte-derived protein with anti-inflammatory, anti-diabetic and anti-atherogenic properties. Adiponectin is also synthesized and secreted by human and murine cardiomyocytes [7,8]. Plasma adiponectin level was reported to be significantly increased in the spontaneously hypertensive, heart failure-prone rats after GLP-1 treatment for 3 months [26]. The present study showed that GLP-1 receptor agonist exenatide significantly increased the level of plasma adiponectin, but had no effect on the myocardial adiponectin levels in diabetic rats. It was reported that GLP-1 receptor agonist exenatide could directly induce adiponectin expression and promote adiponectin secretion in 3 T3-L1 adipocytes [29]. Therefore, the mechanism that GLP-1 increases the plasma adiponectin level may be related to direct stimulation of the synthesis and secretion of adiponectin in adipocytes.

Adiponectin plays its role by binding to its receptors 1 and 2, both are expressed in myocardial tissues [7]. The current study showed that both high and low doses of exenatide treatment could significantly decrease the expression of myocardial adiponectin receptor 1 in diabetic rats. There was no significant difference in the expression of myocardial adiponectin receptor 1 between diabetic rats treated with high and low doses of exenatide. To our knowledge, this is the first report that the expression of myocardial adiponectin receptor 1 was reduced by GLP-1 analogue in diabetic rats. Exenatide may exert its protective effect on the heart of diabetic rats by increasing plasma adiponectin level and decreasing the expression of myocardial adiponectin receptor 1.

Adiponectin can improve glucose metabolism by binding to its receptor and activating the adenosine monophosphate–activated protein kinase (AMPK) signaling pathway [30]. AMPK increases myocardial glucose uptake and utilization through different mechanisms including stimulating the translocation of Glut4 to the sarcolemma in the heart [31]. AMPK deficiency has been reported to be associated with depressed cardiac function in mice [32]. GLP-1 treatment resulted in enhanced phoshorylation of AMPK in normoxic, perfused hearts [33]. Dipeptidyl peptidase IV inhibitor upregulates Glut4 translocation and expression in heart of spontaneously hypertensive rats [34]. This study shows that exenatide treatment increases the level of phosphorylation of AMPK and the mRNA expression of Glut4 in the heart of diabetic rats. The increased adiponectin may partially explain the increased phosphorylation of AMPK and Glut4, which might contribute to the ameliorated heart function in diabetic rats treated with exenatide.

An imbalance between reactive oxygen species generation and antioxidant capacity favors the former causing oxidative stress and oxidative damage [35]. Oxidative stress has been suggested to be involved in the development and progression of diabetes-induced cardiomyopathy [10,11,13,16]. Nicotinamide adenine dinucleotide phosphate (NADPH) oxidase is the main source of reactive oxygen species in the cardiovascular tissues [36]. NADPH oxidase subunits include Nox1-5, p22^phox^, p47^phox^, p67^phox^, and Rac [37]*.* Activation of NAD(P)H oxidase seems to be relevant to the elevated oxidative stress in diabetes [14]. It was reported that GLP-1 decreased reactive oxygen species production and the levels of NADPH oxidase such as p47(phox), gp91(phox), p22(phox) and p40(phox) in high-glucose–induced cardiac microvascular endothelial cells [38]. Glucagon-like peptide-1 receptor (GLP-1R) agonist exendin-4 ameliorated myocardial oxidative stress via suppression of NADPH oxidase 4 with concomitant elevation of antioxidants (SOD-1 and glutathione peroxidase) in type 2 diabetic mice [39]. The present study showed that GLP-1 analogue exenatide reduces the expression of myocardial p22phox, Nox4 and CTGF, increases the expression of myocardial Cu-Zn-SOD, alleviates the myocardial pathological lesion, and improves the heart function. Exenatide can significantly reduce the myocardial oxidative stress and oxidative damage, and produce a protective effect on the heart in diabetic rats.

It is well known that exenatide is effective on reducing body weight as it has been shown to do in models of type 2 diabetes. However, the current study showed that exenatide treatment did not reduce the body weight in a streptozotocin-induced rat model of type 1 diabetes. Therefore, the decreased expressions of myocardial adiponectin receptor and NAPDH subunits by exenatide treatment could not be attributed to the body weight loss in diabetic rats.

## Conlusions

We have shown that long term treatment with exenatide dose-dependently down-regulates the mRNA expression of myocardial p22phox and NOX4, dose-independently down-regulates the expression of myocardial adiponectin receptor 1, and dose-independently up-regulates the level of plasma adiponectin and the expression of myocardial AMPK-α, GLUT4 and Cu-Zn-SOD without lowering blood glucose in a streptozotocin-induced rat model of type 1 diabetes. The increase of myocardial glucose oxidation and the decrease of oxidative stress by exematide may contribute to the improvement of the heart function in diabetic rats. This study may provide the first evidence that GLP-1 receptor agonists may contribute to the prevention of diabetic cardiomyopathy via its regulation effect on adiponectin receptors.

## Abbreviations

NADPH: Nicotinamide adenine dinucleotide phosphate; LVSP: Left ventricular systolic pressure; LVEDP: Left ventricular end-diastolic pressure; NOX4: NADPH oxidase 4; Glut4: Glucose transporter 4; CTGF: Connective tissue growth factor; Cu-Zn-SOD: Copper zinc superoxide dismutase; GLP-1: Glucagon-like peptide-1; ELISA: Enzyme-linked immunosorbent assay; HE: Hematoxylin and eosin; PBS: Phosphate-buffered saline; DAB: Diaminobenzidine; TBS: Tris-buffered saline; AMPK: Adenosine monophosphate–activated protein kinase; adipoR1: Adiponectin receptor 1; PCR: Polymerase chain reaction.

## Competing interests

The authors declare that they have no competing interest.

## Authors’ contributions

Conceived and designed the experiments: ZG. Performed the experiments: WQ YY SD JW JL. Analyzed the data: WQ YY SD JW JL. Wrote the paper: ZG. All authors read and approved the final manuscript.
